# Does It Make Sense to Perform Prostate Magnetic Resonance Imaging in Men with Normal PSA (<4 ng/mL)?

**DOI:** 10.3390/cancers18030423

**Published:** 2026-01-28

**Authors:** Pieter De Visschere, Camille Berquin, Pieter De Backer, Joris Vangeneugden, Eva Donck, Thomas Tailly, Valérie Fonteyne, Sofie Verbeke, Sigi Hendrickx, Nicolaas Lumen, Daan De Maeseneer, Geert Villeirs, Charles Van Praet

**Affiliations:** 1 Department of Radiology and Nuclear Medicine, Ghent University Hospital, 9000 Ghent, Belgium; 2 Faculty of Medicine and Health Sciences, Ghent University, Corneel Heymanslaan 10, 9000 Ghent, Belgium; 3 Department of Urology, Ghent University Hospital, European Reference Network eUROGEN Accredited Center, 9000 Ghent, Belgium; 4 Department of Radiation Therapy, Ghent University Hospital, 9000 Ghent, Belgium; 5 Department of Pathology, Ghent University Hospital, 9000 Ghent, Belgium; 6 Department of Oncology, Ghent University Hospital, 9000 Ghent, Belgium; 7 Department of Oncology, AZ Sint Lucas, 8000 Bruges, Belgium

**Keywords:** prostate cancer, magnetic resonance imaging, PSA, digital rectal examination, biopsy, prostatitis, PI-RADS, benign prostatic disease

## Abstract

**Objective**: We evaluate the performance and relevance of MRI to detect csPC in men with normal PSA. **Methods**: Out of our database of patients referred for prostate MRI, we selected men with PSA < 4 ng/mL for whom histopathology or at least 2 years of clinical follow-up data were available as standard of reference. Subgroup analyses were performed for the patients with PSA < 3 ng/mL, <2 ng/mL, and 2–3.9 ng/mL. The reasons for prostate MRI referral despite their normal PSA level were retrieved by exploring the patients’ files. The prostate MRIs were reported according to the Prostate Imaging and Reporting Data System (PI-RADS), and the overall assessment score was registered. For evaluation of the performance, PI-RADS ≥ 3 was set as a threshold for a positive exam. The patients without PC or only International Society of Urological Pathology (ISUP) grade group 1 PC (Gleason 3+3) were considered as one category having no csPC. The performance of prostate MRI was separately evaluated for detection of ISUP ≥ 2 and for ISUP ≥ 3 csPC. **Results**: A total of 148 men were included, with PSA ranging from 0.42 to 3.99 ng/mL (median 2.95, IQR 1.68–3.50) and age ranging from 36 to 84 years (median 58, IQR 52–66). A total of 74 men (50.0%) had a PSA level < 3 ng/mL, 42 (28.4%) had a PSA level < 2 ng/mL, and 106 (71.6%) had a PSA level of 2–3.9 ng/mL. They were referred for prostate MRI for a wide variety, and usually a combination of, reasons, such as younger age (<60 years in 55.4%, N = 82; <50 years in 17.6%, N = 26), abnormal digital rectal examination in 31.8% of cases (N = 47), suspicious PSA dynamics in 29.7% (N = 44), positive familial history in 27.0% (N = 40), clinical signs of prostatitis in 18.2% (N = 27), suspicious findings on Transrectal Ultrasound (TRUS) in 16.9% (N = 25), hematospermia in 7.4% (N = 11), hematuria in 4.1% (N = 6), incidental hot spot in the prostate on Fluoro-Deoxy-Glucose (FDG) Positron Emission Tomography (PET)–Computed Tomography (CT) in 4.1% (N = 6), lymphadenopathies on CT in 2.7% (N = 4), or severe patient anxiety in 3.4% (N = 5). Overall, ISUP ≥ 2 PC was present in 18.9% (N = 28) of cases, and MRI detected this with a sensitivity of 92.9%, a specificity of 66.7%, and a positive predictive value of 39.4%. ISUP ≥ 3 PC was present in 9.5% (N = 14) of cases, and prostate MRI detected this with a sensitivity of 100%, a specificity of 61.2%, and a positive predictive value of 21.2%. In patients with PSA < 2 ng/mL (N = 42), no csPC was found, but MRI generated false positives in 33.3%. **Conclusions**: Performing prostate MRI in men with normal PSA (<4 ng/mL) seems useful if there are other reasons that increase the clinical suspicion of csPC. In about one-fifth of these patients, csPC is present and MRI has high sensitivity for its detection. Prostate MRI has, however, low positive predictive value in this patient group, and clinicians should be aware of the risk of false-positive MRI. Below a PSA level of 2 ng/mL, no csPC was found and prostate MRI generated only false positives, suggesting limited value in this subgroup.

## Simple Summary

We evaluate the performance and relevance of Magnetic Resonance Imaging (MRI) to detect clinically significant prostate cancer (csPC) in men with normal Prostate Specific Antigen (PSA), defined as PSA < 4 ng/mL. A total of 148 men were included, and subgroup analyses were performed for the patients with PSA < 3 ng/mL, <2 ng/mL, and 2–3.9 ng/mL. Despite their normal PSA level, they were referred for prostate MRI for a variety of reasons such as younger age, abnormal digital rectal examination, positive familial history, or findings on other imaging suggestive of prostate cancer. Overall, csPC was present in 18.9% of cases, and MRI detected this with a sensitivity of 92.9%. Performing prostate MRI in men with normal PSA level seems useful if there are other reasons that increase the clinical suspicion of csPC, but below a PSA level of 2 ng/mL, no csPC was found and prostate MRI generated only false positives, suggesting limited value in this subgroup.

## 1. Introduction

Early detection of PC is performed by means of serum PSA testing and digital rectal examination (DRE). Current European Association of Urology (EAU) guidelines propose PSA testing for early detection of PC in men with a life expectancy exceeding 15 years [1,2]. Screening with PSA and DRE is associated with a significant increase in PC detection, with a shift towards more localized and low-grade tumors, resulting in a 20% PC-specific mortality reduction [3]. Organized population screening for PC with PSA is currently not recommended due to concerns about overdiagnosis and overtreatment, but individual (opportunistic) testing it is commonly performed in men who are well informed about the risks and benefits [4].

In patients without risk factors, a cut-off value of 3 or 4 ng/mL is used to decide whether to perform a prostate biopsy [2], although it is well recognized that PC may still be present below these PSA thresholds, with a substantial proportion being clinically significant [5,6,7,8,9]. In a study by Thompson et al., the prevalence of PC among 2950 men with a PSA level ≤ 4 ng/mL was 15.2%, although most cancers were low-grade with a prevalence of 2.2% of ISUP ≥ 2 PC [6]. In men with PSA values between 3.1 and 4 ng/mL, PC was present in 26.9%, and 6.7% had ISUP ≥ 2 PC [6]. In clinical practice, the optimal PSA threshold for the diagnosis of PC and indication for prostate biopsy should offer a reasonable balance, as a low threshold will result in excessive sampling of normal tissue and detection of indolent PC (overdiagnosis), while a high threshold holds the risk of missing csPC [7,8].

MRI of the prostate has become an established tool to detect csPC and avoid overdiagnosis of indolent PC in men with increased PSA [1,10]. Prostate MRI may be multiparametric (mpMRI) or biparametric (bpMRI) [11,12,13], and is preferably scanned and reported according to the PI-RADS guidelines [14,15,16]. According to the EAU guidelines, prostate MRI is currently indicated prior to prostate biopsy. In the case of a positive MRI (PI-RADS 3-5), prostate biopsy is indicated and can be targeted to the MRI lesion. In the case of a negative MRI (PI-RADS 1-2), prostate biopsy can be omitted as there is a (very) low chance of detecting csPC [1]. Prostate MRI has proven to be accurate in patients with elevated PSA, but it is currently unknown how many csPCs MRI could identify in the lower PSA range. This may be relevant in patients presenting with normal PSA but increased clinical suspicion of PC because of positive familial risk, abnormal DRE, younger age, or suspicious findings on other imaging (TRUS, FDG-PET-CT, pelvic adenopathy on CT, or MRI performed for other reasons), as well as in patients treated with immunosuppressive drugs or having clinical prostate-related symptoms. Therefore, in this study, we aimed to evaluate the performance and relevance of prostate MRI to detect csPC in men with normal PSA levels. We hypothesized that prostate MRI can detect a meaningful proportion of csPC even in men with normal PSA levels, particularly when other risk factors are present.

## 2. Materials and Methods

Out of our database of patients who were referred for diagnostic prostate MRI at our tertiary referral center between November 2012 and December 2020, we selected men with PSA levels < 4 ng/mL at the time of their MRI for whom histopathology or at least 2 years of subsequent clinical follow-up was available as standard of reference. Subgroup analyses were performed in men with PSA < 3 ng/mL, <2 ng/mL, and 2–3.9 ng/mL. Patients who had been treated previously for PC or who had had transurethral resection (TURP) as treatment for benign prostatic hyperplasia in the past were excluded. The indication(s) for prostate MRI referral were retrieved from the digital patient files.

All MRI scans were performed on a 3.0 Tesla scanner (Magnetom Trio, Siemens Healthineers^TM^, Erlangen, Germany) without endorectal coil and consisted of at least T2-weighted imaging and diffusion-weighted imaging (DWI) (bpMRI), occasionally supplemented with dynamic contrast-enhanced imaging (DCE) (mpMRI). In all patients, butylscopalamine bromide (Buscopan^®^, Opella Healthcare, Berkshire, UK) was given intravenously before the examination to suppress bowel motility. All exams were analyzed on a PACS workstation (Agfa Impax^TM^, Mortsel, Belgium). Two radiologists with 10 and 15 years of experience (PDV and GV) evaluated the MRI scans and assigned an overall assessment score according to the PI-RADS scoring system [15]. For patients with multiple lesions, the overall assessment score of the lesion with the highest score was used. To evaluate the performance of prostate MRI, the scores were dichotomized with PI-RADS ≥ 3 set as threshold for a positive exam.

Clinical follow-up data consisted of histopathology from prostate biopsy, TURP, or radical prostatectomy, and follow-up of at least 2 years with repetitive PSA measurements, repeat prostate biopsy, and interval prostate MRI performed at the discretion of the referring clinician. Prostate biopsies consisted of TRUS or transperineal-ultrasound-guided systematic biopsies, supplemented with targeted cores to the suspicious areas in the case of PI-RADS ≥ 3 lesion on prostate MRI, by using a cognitive approach, TRUS-MRI fusion, or in-bore targeted biopsy. Patients with PC were classified according to the 5-point grading system of the ISUP. If after 2 years of follow-up prostate biopsies remained negative and PSA remained stable or decreased spontaneously without suspicious findings on subsequent imaging, the patient was considered to have no csPC. Patients with ISUP ≥ 2 were categorized as having csPC. The performance of prostate MRI was evaluated for detection of ISUP ≥ 2 and ISUP ≥ 3 PC. For statistical analyses, a software package (SPSS for Windows™, version 27.0; SPSS, Chicago, IL, USA) was used. All patients signed an informed consent form, and the study was approved by the hospital’s ethics committee (EC2011/495 with amendment on 18 November 2015).

## 3. Results

**Figure 1:**
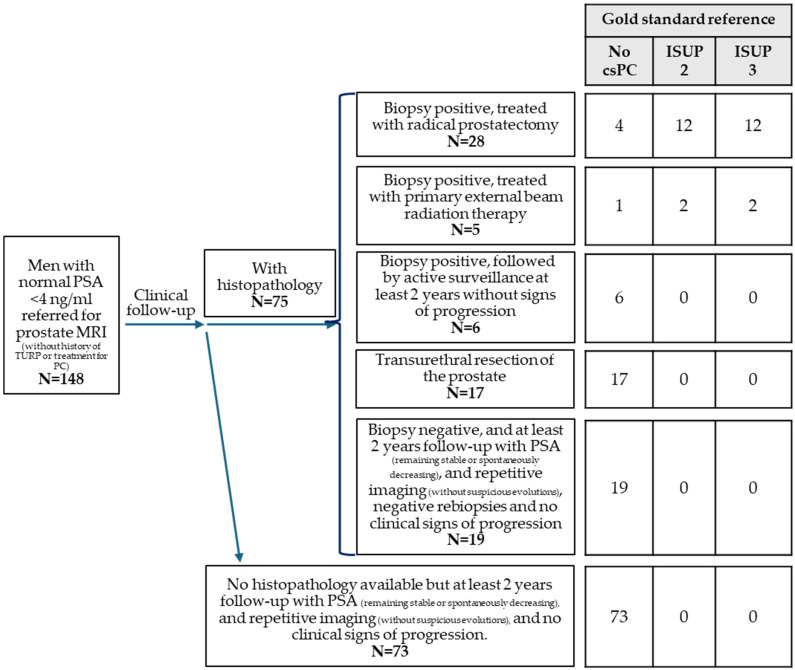
Clinical follow-up after prostate MRI in men with normal PSA < 4 ng/mL.

A total of 148 men met the inclusion criteria of this study. The patients’ ages ranged from 36 to 84 years (median 58, IQR 52–66). The PSA ranged from 0.42 to 3.99 ng/mL (median 2.95, IQR 1.68–3.50) and the PSA density ranged from 0.01 to 0.24 ng/ml^2^ (median 0.07, IQR 0.04–0.10). A total of 74 patients (50.0%) had a PSA level < 3 ng/mL, 42 (28.4%) had a PSA level < 2 ng/mL, and 106 (71.6%) had a PSA level of 2–3.9 ng/mL. In 106 (71.6%) patients, bpMRI was performed, while mpMRI was performed in 42 (28.4%). The standard of reference was based on histopathology in 75 patients (50.7%), of which 30 (38.5%) were from prostate biopsy (followed by active surveillance in 6 patients, external beam radiotherapy in 5 patients, and routine clinical follow-up in 19 patients), 17 (21.8%) were from TURP, and 28 (35.9%) were from radical prostatectomy. In 81.1% of the patients (N = 120), no csPC was found during the 2 years of follow-up after prostate MRI (Figure 1).

In 18.9% of the patients (N = 28), ISUP ≥ 2 PC was detected, and in 9.5% (N = 14) ISUP ≥ 3 PC was detected. All these tumors were detected in the subgroup of patients with PSA between 2 and 3.9 (N = 106), as in the 42 patients with PSA < 2 ng/mL, no csPC was found. In the 106 patients with PSA between 2 and 3.9, ISUP ≥ 2 PC was present in 26.4% (N = 28) and ISUP ≥ 3 PC in 13.2% (N = 14). Of the 74 patients with PSA < 3 ng/mL, 93.2% (N = 69) had no csPC, in 6.8% (N = 5) ISUP ≥2 PC was present, and in only 4.1% (N = 3) ISUP ≥ 3 PC was found (Table 1).

The patients were referred for prostate MRI despite a normal PSA for a variety of reasons and most often a combination of multiple factors. PSA-related features played a role, such as PSA ≥ 3 ng/mL (but below 4 ng/mL), which was the case in 50.0% of patients (N = 74). In this group, ISUP ≥ 2 PC was present in 31.1% of patients (N = 23) and ISUP ≥ 3 PC was present in 14.9% (N = 11). PSA dynamics (rapidly rising PSA) were present in 29.7% (N = 44) of patients and were associated with ISUP ≥ 3 PC in 13.6%. PSA density > 0.15 was observed in 4.1% of patients (N = 6). Age was another reason for referral as 55.4% (N = 82) of the patients were <60 years and 17.6% (N = 26) of the patients were <50 years. Higher risk of PC was suspected in patients with familial history of PC (at least one first- or second-degree relative with PC), which was the case in 27.0% (N = 40) of our patient group; however, no ISUP ≥3 PC was found in these patients, and ISUP 2 PC was found in only three patients (7.5%). In total, 3.4% (N = 5) of patients were referred for MRI because of HIV or immunosuppressive treatments but no csPC was present. Clinical findings such as abnormal DRE were a reason for prostate MRI referral. This was present in about one-third of the patients (31.8%, N = 47) and it was associated with ISUP ≥ 2 PC in 34.4% of patients (N = 16) and with ISUP ≥ 3 PC in 21.3% (N = 10). Hematospermia was a reason for prostate MRI in 7.4% of patients (N = 11), hematuria in 4.1% (N = 6), and clinical signs of prostatitis in 18.2% (N = 27). In these patients, ISUP ≥ 2 PC was present in 9.1%, 16.7%, and 7.4% of cases, respectively. Findings on other imaging methods were another reason for prostate MRI referral. A suspicious lesion on TRUS was reported in 16.9% (N = 25) of patients and was associated with ISUP ≥ 2 PC in 36.0% (N = 9) and with ISUP ≥ 3 PC in 24.0% (N = 9). In 4.1% (N = 6) of patients, a hot spot in the prostate was incidentally detected on FDG-PET-CT performed for other reasons (lymphoma, melanoma, other cancers) but no ISUP ≥ 3 PC was found, and only one ISUP 2 PC was found. The FDG uptake was caused by prostatitis in the other patients. In four patients (2.7%), prostate MRI was performed because pelvic lymphadenopathies were incidentally observed on CT but PC was not detected in any of them. In five patients (3.4%), patient anxiety was reported as the reason to perform prostate MRI to further reassure the patient about the absence of csPC although the PSA level was normal, and no PC was found in these patients. In 48 patients (32.4%), prostate MRI was performed for LUTS (lower urinary tract symptoms), although mainly to exclude unexpected PC in the view of planned surgery. In these patients, ISUP ≥ 2 PC was present in nine cases (18.8%) and ISUP ≥ 3 PC in six (12.5%). In most patients, a combination of the above-mentioned factors accumulated into the decision to perform prostate MRI. A summary is presented in Table 2, and the detailed patient information is available in Supplementary Table S1.

In the total study population of 148 patients with PSA level < 4 ng/mL, prostate MRI was positive (PI-RADS ≥ 3) in 44.6% (N = 66) and negative in 55.4% (N = 82). In all patients with ISUP ≥ 3 PC (N = 14, 9.5%), MRI was positive. Although none of the men with PSA < 2 ng/mL (N = 42) had csPC, MRI was nonetheless scored positive in 33.3% (N = 14), of which PI-RADS 4 was detected in 7.1% (N = 3) and PI-RADS 5 in one patient (2.4%) who was histopathologically diagnosed with granulomatous prostatitis.

Table 3 shows the performance of prostate MRI (PI-RADS ≥3 considered positive) for detection of ISUP ≥ 2 and ISUP ≥ 3 PC in patients with normal PSA levels. For detecting csPC (ISUP ≥ 2) in the patients with a PSA level < 4 ng/mL, prostate MRI had a sensitivity of 92.9%, a specificity of 66.7%, a positive predictive value (PPV) of 39.4%, a negative predictive value (NPV) of 97.6%, and an accuracy of 71.6%. For detection of csPC (ISUP ≥ 2) in the patients with a PSA level < 2 ng/mL, the sensitivity was unmeasurable and the PPV was zero due to the absence of csPC in this subgroup.

In Table 1, the PPV of prostate MRI (PI-RADS ≥ 3 considered positive) for detection of ISUP ≥ 2 is demonstrated for each individual referral reason. PPV appeared to be higher if PSA was ≥3 ng/mL (54.8%), if PSA dynamics were suspicious (55.0%), if DRE was suspicious (57.1%), or if TRUS was suspicious (56.3%). PPV of prostate MRI was zero in patients with pelvic lymphadenectomy, severe anxiety, and immunosuppressive treatments or HIV.

## 4. Discussion

In this study we assessed the performance and relevance of prostate MRI in patients with normal PSA, which we defined as PSA < 4 ng/mL. Our study demonstrates that in such patients, ISUP ≥ 2 PC was present in about one-fifth (18.9%) and ISUP ≥ 3 PC in about one in ten (9.5%) and that prostate MRI detected these cancers with very high sensitivity (92.9% and 100%, respectively) but moderate specificity (66.7% and 61.2%, respectively). The positive predictive value was very low (39.4% and 21.2%, respectively) and this has important implications because clinicians have to be aware of the risk of false-positive MRI in this patient group with normal PSA and low likelihood of csPC. In patients with PSA < 2 ng/mL, even though no csPC was present, prostate MRI generated false positives (PI-RADS ≥ 3) in 33.3% of cases. Our findings suggest that performing prostate MRI nevertheless may be useful in patients with normal PSA (<4 ng/mL) if there are other reasons that increase the clinical suspicion of csPC. A first such reason is age, as a slightly elevated PSA (between 3 and 4 ng/mL) may be more worrisome in very young men than in older patients. In our study, for uniformity and clarity, we have chosen 4 ng/mL as the general threshold for normal PSA, and decided not to use an age-adjusted PSA level because owing to the modest sensitivity of PSA, international guidelines have been unable to provide a clear threshold recommendation [17,18].

A second reason is suspicious PSA dynamics or PSA density. A rapidly rising PSA in a short time period or a PSA rise of >0.75 ng/mL per year may indeed be worrisome, even though the level remains below the normal thresholds. PSA density was normal (≤0.15) in almost all of our men (95.9%, N = 142), which is not surprising given the (very) low PSA values. PSA density is, after all, a parameter to evaluate whether an elevated PSA is caused by benign prostatic hyperplasia or PC [19], and it is thus not actually intended for use in patients with normal PSA. This is confirmed in our study, as in the six patients with PSA density > 0.15, no csPC was found in five (83.3%) and only one had ISUP 2 PC.

A third reason for performing prostate MRI in patients with normal PSA is suspicious DRE, which appeared to be an important and relevant risk factor in our study as it was associated with ISUP ≥ 2 PC in 34.0% of patients and with ISUP ≥ 3 in 21.3%. Nevertheless, many studies showed that the value of DRE is limited when PSA is normal [17,20,21,22]. In a study comparing the biopsy rate and diagnostic yield in patients with suspicious DRE but normal PSA versus patients with elevated PSA (irrespective of DRE findings), Richards et al. [17] found that an abnormal DRE with normal PSA was less likely to indicate PC than with a raised PSA. This is in line with the study of Sajjad et al. [23], showing that csPC detection rate is higher in patients with elevated PSA than in patients with abnormal DRE and normal PSA. Richards et al. reported that 58% of patients with abnormal DRE had a normal posterior prostate appearance on MRI, demonstrating the low accuracy of DRE [17].

A fourth reason for performing prostate MRI in patients with normal PSA is suspicious TRUS, and it appears important as it was associated with ISUP ≥ 2 PC in 36.0% of patients and with ISUP ≥ 3 in 24.0% in our study.

Familial risk of PC is a fifth and established risk factor. There are inherited genetic factors such as BRCA1 or 2 mutations, but the risk of PC also increases with the number and closeness of affected relatives. Having many relatives with PC may cause patient anxiety and this also appeared to be a reason to perform prostate MRI irrespective of the PSA level in our study. Prostate MRI may be used mainly to exclude rather than to detect csPC in these cases to reassure the very worried patients.

Other factors that play a role in prostate MRI referral in patients with normal PSA are hematospermia, hematuria, pelvic pain, or clinical signs of prostatitis. A prostate MRI may be indicated to find the cause of the complaints in these patients, independently of the PSA level. An incidental hot spot in the prostate on whole-body FDG-PET-CT was also a reason for performing prostate MRI in our study. The sensitivity of FDG-PET-CT for detecting PC, however, is low, and usually the incidental hot spot in the prostate is caused by (subclinical) prostatitis [24]. Similarly, an incidental pelvic adenopathy on CT or MRI was a reason for performing prostate MRI, but we found no csPC as cause of the the lymph nodes. Some patients with clinical symptoms of LUTS in our study were referred for prostate MRI. Although LUTS is not a risk factor for PC, it may be useful to perform prostate MRI if surgery (TURP, adenomectomy, etc.) is planned to avoid unexpected csPC. As mentioned before, there is usually not a single reason for performing prostate MRI, but a combination of multiple factors motivates the decision. Although it appeared that some factors occurred more frequently together (e.g., abnormal DRE and suspicious TRUS in the same region), we observed that the combination of factors was different for every individual patient, and therefore, no relevant subgroups of combinations could be identified.

Our PC detection rates in men with normal PSA are consistent with the literature. Thompson et al. [6] reported the presence of PC in 15.2% of asymptomatic men with PSA < 4 ng/mL, of which 14.9% were ISUP ≥ 2 PC. In our study of selected patients, similarly, ISUP ≥ 2 PC was present in 18.9% in men with PSA < 4 ng/mL. In the study by Thompson et al., PC was present in 26.9% of men with PSA 3.1–4.0 ng/mL (6.7% ISUP ≥ 2 PC) and in 23.9% of men with PSA 2.1–3.0 ng/mL (4.6% ISUP ≥ 2 PC), similar to our study. They found, however, PC in 17% of men with PSA 1.1–2.0 ng/mL, in 10.1% of men with PSA 0.6–1.0 ng/mL, and in 6.6% of men with PSA < 0.5 ng/mL (2.0%, 1.0%, and 0.8% ISUP ≥ 2 PC, respectively) [6]. According to these data, it may appear that no safe lower PSA limit exits below which no csPC occurs and that ‘normal’ PSA maybe does not exist, but in our study, we did not find any csPC in the 42 patients with PSA levels < 2 ng/mL. Since in clinical practice, a PSA threshold of 3 or 4 ng/mL is often used as a trigger to perform a biopsy, we arbitrarily chose for our study a threshold of 4 ng/mL as the upper limit for what could be considered as ‘normal’ PSA and assessed the MRI performance in the subgroups of patients with PSA < 3, PSA < 2, and PSA between 2 and 3.9 ng/mL. Okotie et al. [5] reported PC in 18% of men with PSA < 4 ng/mL. They found PC with ISUP ≥ 2 in 10%, 22%, 14%, and 35% of patients at PSA levels of <1.0, 1.0–2.0, 2.0–3.0, and 3.0–4.0 ng/mL, respectively, but, importantly, they included only patients with abnormal DRE. In our study, the subgroup of patients with abnormal DRE and PSA < 4.0 ng/mL consisted of 47 patients (31.7% of the patient group) and ISUP ≥ 2 was present in 34.0%. We found ISUP ≥ 2 in 0%, 0%, 35.7%, and 52.4% in the same PSA groups of <1.0, 1.0–2.0, 2.0–3.0, and 3.0–4.0 ng/mL, respectively. PSA alone thus appears insufficient to decide to biopsy and other parameters such as family risk and DRE should be considered, as well as prostate MRI.

The sensitivities of prostate MRI in patients with PSA < 4 ng/mL appeared to be similar to the ones reported in the literature in patients with elevated PSA (≥4 ng/mL), around 90% for ISUP ≥ 2 PC, but the specificities were lower, about 65% compared to 80% [13]. This can be explained by the low prevalence of csPC in our preselected group of patients with normal PSA, even though some of them had high familial risk (27.0%) and/or had abnormal DRE (31.8%). The low pre-test probability of csPC in these patients explains the very high negative predictive values observed in our study at the expense of the low positive predictive values of only 20.8% in patients with PSA < 3 ng/mL and zero in patients with PSA < 2 ng/mL due to the absence of csPC in the latter patient group.

Al-Monajjed et al. performed an analysis of the mpMRI findings in a screening study of healthy younger men (47–52 years) with PSA < 3 ng/mL [18]. They included 47 men and observed PI-RADS 1 in 2.1%, PI-RADS 2 in 53%, PI-RADS 3 in 45%, and PI-RADS 4 or 5 in 0%. Similarly, in our study, we found PI-RADS 1 in 7.7% (N = 2), PI-RADS 2 in 42.3% (N = 11), PI-RADS 3 in 42.3%, PI-RADS 4 in 3.8% (N = 1), and PI-RADS 5 in 3.8% (N = 1) of patients younger than 50 years (N = 26). After 2 years of follow-up, Al-Monajjed et al. still did not detect any PC in their patient group, whereas we found three ISUP 2 PC (11.5%) and one ISUP 4 (3.8%). This difference can be explained by the fact that their patients were asymptomatic men in a screening setting, while our patients had at least one other reason for suspicion of PC. Other studies have explored the use of prostate MRI as a first-line screening test for PC, using prostate MRI regardless of their PSA values and potentially even replacing PSA testing entirely (e.g., IP1-PROSTAGRAM, ReIMAGINE, and PROSA trials [9,25,26]). Nam et al. [27] explored the feasibility of prostate MRI as a first-line screening test for PC in an unselected sample of the general population. They used PI-RADS ≥ 4 as the threshold for a positive MRI exam. Among the men with PSA < 4 ng/mL, this resulted in a PPV of 66.7% and an NPV of 85.7%. With the same threshold set in our study, this would result in a PPV of 61.9% and an NPV of 98.1% for detection of ISUP ≥ 2 PC. The results of our study, however, cannot be extrapolated to the screening setting since most of our patients underwent prostate MRI because of increased suspicion of csPC based on other reasons than PSA. In future research, prospective studies are needed in volunteers to compare PSA and prostate MRI independently with regard to csPC detection and avoidance of insignificant PC [28].

There are several limitations to our study. The first limitation is the retrospective nature and heterogeneity of our study population, even though the common factor was a prostate MRI with a normal PSA level and referral for a variety of reasons. Most patients were referred for a combination of reasons, resulting in a complex mixture of factors. Ideally, they would each be referred for a single reason and thus well-defined independent subgroups could have been assessed. The heterogeneity of the study population also resulted in smaller sample size of some subgroups, such as the subgroup of patients with PSA < 2 ng/mL, which consisted of only 42 patients. Secondly, also due to the retrospective nature of the study, clinical features were based on explicit mentioning in the patient files. They were not standardized and were performed by different clinicians, which may have caused subjective differences due to high examiner dependency [17]. Nevertheless, we think that our study provides relevant data as the heterogeneous patient group reflects real-world clinical practice.

Third, we did not have histopathology as the gold standard in 49.3% (N = 73) of our patients, because the PSA remained stable or decreased spontaneously, and no suspicious findings were observed on imaging in the following 2 years. This was mainly the case in patients with a negative MRI (N = 82) and normal PSA, in whom a biopsy was not performed (N = 59). In Supplementary Table S2 the availability of histopathology (biopsy, TURP, radical prostatectomy) in our patients in relation to MRI result is shown. We separately assessed the performance of prostate MRI in the subgroup of patients with available histopathology (N = 75) and compared this with the total study population but found similar results. In Supplementary Table S3 the performance parameters in patients with and without availability of histopathology are provided. In our cohort of men with normal PSA, we considered a follow-up period of 2 years—with PSA remaining stable or spontaneously decreasing, repetitive imaging (without suspicious evolutions), and no clinical signs of progression—as even more convincing to exclude the presence of csPC than a prostate biopsy, of which it is well known that it may miss up to 30 to 40% of cancers [29,30]. Therefore, in our opinion, the risk of verification bias is limited. We adopted this follow-up period of 2 years as a longer follow-up would increase the chance of detecting newly formed PC that was not present at the time of the initial MRI. A last limitation was that the radiologists reporting the MRI were not blinded to the patients’ PSA, which can be a source of confirmation bias. We did not perform a second blind reading because the radiologists were highly experienced and strictly followed the PI-RADS guidelines, which are theoretically independent of clinical information. Nevertheless, knowledge of the low PSA value or the other reasons for clinical suspicion of PC may have influenced the MRI reporting in doubtful cases.

## 5. Conclusions

In this study we assessed the performance and relevance of prostate MRI in patients with normal PSA, which we defined as PSA < 4 ng/mL. Despite the normal PSA, they were referred for a variety and often a combination of other reasons that increased the clinical suspicion of csPC. Performing prostate MRI in these men seems useful because in about one-fifth of the patients csPC is present and MRI has high sensitivity for its detection. Prostate MRI, however, has very low positive predictive value in this patient group and below a PSA level of 2 ng/mL, no csPC was found and prostate MRI generated only false positives, suggesting limited value in this subgroup. The main limitations of our study are its retrospective nature, the heterogeneity of the study population, and the small subgroup analysis. The implications of our findings for clinical practice include that performing prostate MRI in patients with normal PSA makes most sense if the PSA level is between 2 and 3.9 ng/mL, and that clinicians have to be aware of the high risk of false-positive MRI. If the PSA level is below the threshold of 2 ng/mL, clinical follow-up might be better than performing prostate MRI.

## Figures and Tables

**Table 1:** MRI PI-RADS score and standard of reference in patients with PSA <4, <3, <2, and 2–3.9 ng/mL.

	MRI Score	PI-RADS 1	PI-RADS 2	PI-RADS 3	PI-RADS 4	PI-RADS 5	MRI Negative (PI-RADS 1 or 2)	MRI Positive (PI-RADS ≥ 3)	Total
PSA < 4 (all patients)	N (%)	14 (9.5%)	68 (45.9%)	32 (21.6%)	22 (14.9%)	12 (8.1%)	82 (55.4%)	66 (44.6%)	148
	No csPC ^1^	14 (100%)	66 (97.1%)	24 (75.0%)	13 (59.1%)	3 (25.0%)	80 (97.6%)	40 (60.6%)	120 (81.1%)
	ISUP ≥ 2	0	2 (2.9%)	8 (25.0%)	9 (40.9%)	9 (75.0%)	2 (2.4%)	26 (39.4%)	28 (18.9%)
	ISUP ≥ 3	0	0	5 (15.6%)	4 (18.2%)	5 (41.7%)	0	14 (21.2%)	14 (9.5%)
PSA < 3 subgroup	N (%)	13 (17.6%)	37 (50.0%)	14 (18.9%)	7 (9.5%)	3 (4.1%)	50 (67.6%)	24 (32.4%)	74
	No csPC ^1^	13 (100%)	37 (100%)	12 (85.7%)	5 (71.4%)	2 (66.7%)	50 (100%)	19 (79.2%)	69 (93.2%)
	ISUP ≥ 2	0	0	2 (14.3%)	2 (28.6%)	1 (33.3%)	0	5 (20.8%)	5 (6.8%)
	ISUP ≥ 3	0	0	2 (14.3%)	1 (14.3%)	0	0	3 (12.5%)	3 (4.1%)
PSA < 2 subgroup	N (%)	7 (16.7%)	21 (50.0%)	10 (23.8%)	3 (7.1%)	1 (2.4%)	28 (66.7%)	14 (33.3%)	42
	No csPC ^1^	7 (100%)	21 (100%)	10 (100%)	3 (100%)	1 (100%)	28 (100%)	14 (100%)	42 (100%)
	ISUP ≥ 2	0	0	0	0	0	0	0	0
	ISUP ≥ 3	0	0	0	0	0	0	0	0
PSA 2–3.9 subgroup	N (%)	7 (6.6%)	47 (44.3%)	22 (20.8%)	19 (17.9%)	11 (10.4%)	54 (50.9%)	52 (49.18%)	106
	no csPC ^1^	7 (100%)	45 (95.7%)	14 (63.6%)	10 (52.6%)	2 (18.2%)	52 (96.3%)	26 (50.0%)	78 (73.6%)
	ISUP ≥ 2	0	2 (4.3%)	8 (36.4%)	9 (47.4%)	9 (81.8%)	2 (3.7%)	26 (50.0%)	28 (26.4%)
	ISUP ≥ 3	0	0	5 (22.7%)	4 (21.1%)	5 (45.5%)	0	14 (26.9%)	14 (13.2%)

^1^ No csPC = no clinically significant prostate cancer; this includes patients without PC and patients with ISUP 1 PC.

**Table 2:** Reasons for performing prostate MRI despite normal PSA, with MRI results and gold-standard reference *.

	All 148 Patients		MRI Positive (PI-RADS ≥ 3)		Gold-Standard Reference			PPV of a Positive MRI for ISUP ≥ 2
	N	%	N	%	No csPC ^1^	% ISUP ≥ 2	% ISUP ≥ 3	
PSA ≥ 3 ng/mL (but below 4 ng/mL, i.e., 3–3.9 ng/mL)	74	50.0%	42	56.8%	51 (68.9%)	23 (31.1%)	11 (14.9%)	54.8%
PSA dynamics suspicious	44	29.7%	20	45.5%	33 (75.0%)	11 (25.0%)	6 (13.6%)	55.0%
PSA density > 0.15	6	4.1%	4	66.7%	5 (83.3%)	1 (16.7%)	0	25.0%
Age < 50 years	26	17.6%	13	50.0%	22 (84.6%)	4 (15.4%)	1 (3.8%)	30.8%
Age < 60 years	82	55.4%	38	46.3%	67 (81.7%)	15 (18.3%)	6 (7.3%)	39.5%
Familial history positive (prostate cancer in at least one first- or second-degree relative)	40	27.0%	12	30.0%	37 (92.5%)	3 (7.5%)	0	25.0%
Immunosuppressive treatments or HIV	5	3.4%	1	20.0%	5 (100%)	0	0	0
Severe patient anxiety	5	3.4%	0	0	5 (100%)	0	0	0
DRE suspicious	47	31.8%	28	59.6%	31 (66.0%)	16 (34.0%)	10 (21.3%)	57.1%
TRUS suspicious	25	16.9%	16	64.0%	16 (64.0%)	9 (36.0%)	6 (24.0%)	56.3%
Hematospermia	11	7.4%	6	54.5%	10 (90.9%)	1 (9.1%)	1 (9.1%)	16.7%
Hematuria	6	4.1%	2	33.3%	5 (83.3%)	1 (16.7%)	1 (16.7%)	50.0%
Clinical signs of prostatitis	27	18.2%	12	44.4%	25 (92.6%)	2 (7.4%)	2 (7.4%)	16.7%
LUTS	48	32.4%	20	41.7%	39 (81.3%)	9 (18.8%)	6 (12.5%)	45.0%
Pelvic lymphadenopathies on CT	4	2.7%	0	0	4 (100%)	0	0	0
Hot spot in the prostate on FDG-PET-CT	6	4.1%	3	50.0%	5 (83.3%)	1 (16.7%)	0	33.3%

^1^ No csPC = no clinically significant prostate cancer; this includes patients without PC and patients with ISUP 1 PC. * In most patients, a combination of multiple factors resulted in prostate MRI referral

**Table 3:** Performance of prostate MRI (PI-RADS ≥ 3 considered positive) for detection of ISUP ≥ 2 and ISUP ≥ 3 prostate cancer in patients with normal PSA (<4, <3, <2, and 2–3.9).

		PSA < 4 (All Patients)	PSA < 3 Subgroup	PSA < 2 Subgroup	PSA 2–3.9 Subgroup
for detection of ISUP ≥ 2 prostate cancer	sensitivity	92.9%	100%	/	92.9%
	specificity	66.7%	72.5%	66.7%	66.7%
	PPV	39.4%	20.8%	0.0%	50.0%
	NPV	97.6%	100%	100%	96.3%
	accuracy	71.6%	74.3%	66.7%	73.6%
for detection of ISUP ≥ 3 prostate cancer	sensitivity	100%	100%	/	100%
	specificity	61.2%	70.4%	66.7%	58.7%
	PPV	21.2%	12.5%	/	26.9%
	NPV	100%	100%	100%	100%
	accuracy	64.9%	71.6%	66.7%	64.2%

## Data Availability

The raw data supporting the conclusions of this article will be made available by the authors on request.

## Supplementary Materials

The following supporting information can be downloaded at https://www.mdpi.com/article/10.3390/cancers18030423/s1. Table S1: Overview of the clinical data of the 148 patients included in the study; Table S2: Availability of histopathology in relation with MRI result; Table S3: Performance in patients with and without availability of histopathology.

## Abbreviations

The following abbreviations are used in this manuscript:

PC	Prostate cancer
csPC	Clinically significant prostate cancer
PSA	Prostate Specific Antigen
DRE	Digital rectal examination
EAU	European Association of Urology
TRUS	Transrectal Ultrasound
MRI	Magnetic Resonance Imaging
bpMRI	Biparametric MRI
mpMRI	Multiparametric MRI
DWI	Diffusion-weighted imaging
DCE	Dynamic contrast-enhanced imaging
PI-RADS	Prostate Imaging and Reporting Data System
PET-CT	Positron Emission Tomography—Computed Tomography
TURP	Transurethral Resection of the Prostate
ISUP	International Society of Urological Pathology

## References

[1] EAU Guidelines. Edn. Presented at the EAU Annual Congress Madrid 2025. https://uroweb.org/guidelines/prostate-cancer.

[2] Catalona W.J., Smith D.S., Ratliff T.L., Dodds K.M., Coplen D.E., Yuan J.J., Petros J.A., Andriole G.L. (1991). Measurement of prostate-specific antigen in serum as a screening test for prostate cancer. N. Engl. J. Med..

[3] Hugosson J., Roobol M.J., Mansson M., Tammela T.L.J., Zappa M., Nelen V., Kwiatkowski M., Lujan M., Carlsson S.V., Talala K.M. (2019). A 16-yr Follow-up of the European Randomized study of Screening for Prostate Cancer. Eur. Urol..

[4] Lumen N., Fonteyne V., De Meerleert G., Ost P., Villeirs G., Mottrie A., De Visschere P., De Troyer B., Oosterlinck W. (2012). Population screening for prostate cancer: An overview of available studies and meta-analysis. Int. J. Urol. Off. J. Jpn. Urol. Assoc..

[5] Okotie O.T., Roehl K.A., Han M., Loeb S., Gashti S.N., Catalona W.J. (2007). Characteristics of prostate cancer detected by digital rectal examination only. Urology.

[6] Thompson I.M., Pauler D.K., Goodman P.J., Tangen C.M., Lucia M.S., Parnes H.L., Minasian L.M., Ford L.G., Lippman S.M., Crawford E.D. (2004). Prevalence of Prostate Cancer among Men with a Prostate-Specific Antigen Level ≤4.0 ng per Milliliter. N. Engl. J. Med..

[7] Schroder F.H., Roobol M.J. (2009). Defining the optimal prostate-specific antigen threshold for the diagnosis of prostate cancer. Curr. Opin. Urol..

[8] Thompson I.M., Ankerst D.P., Chi C., Lucia M.S., Goodman P.J., Crowley J.J., Parnes H.L., Coltman C.A. (2005). Operating characteristics of prostate-specific antigen in men with an initial PSA level of 3.0 ng/ml or lower. JAMA.

[9] Eldred-Evans D., Burak P., Connor M.J., Day E., Evans M., Fiorentino F., Gammon M., Hosking-Jervis F., Klimowska-Nassar N., McGuire W. (2021). Population-Based Prostate Cancer Screening With Magnetic Resonance Imaging or Ultrasonography: The IP1-PROSTAGRAM Study. JAMA Oncol..

[10] Mottet N., van den Bergh R.C.N., Briers E., Van den Broeck T., Cumberbatch M.G., De Santis M., Fanti S., Fossati N., Gandaglia G., Gillessen S. (2021). EAU-EANM-ESTRO-ESUR-SIOG Guidelines on Prostate Cancer-2020 Update. Part 1: Screening, Diagnosis, and Local Treatment with Curative Intent. Eur. Urol..

[11] Boesen L., Norgaard N., Logager V., Balslev I., Bisbjerg R., Thestrup K.C., Winther M.D., Jakobsen H., Thomsen H.S. (2018). Assessment of the Diagnostic Accuracy of Biparametric Magnetic Resonance Imaging for Prostate Cancer in Biopsy-Naive Men: The Biparametric MRI for Detection of Prostate Cancer (BIDOC) Study. JAMA Netw. Open.

[12] De Visschere P., Lumen N., Ost P., Decaestecker K., Pattyn E., Villeirs G. (2017). Dynamic contrast-enhanced imaging has limited added value over T2-weighted imaging and diffusion-weighted imaging when using PI-RADSv2 for diagnosis of clinically significant prostate cancer in patients with elevated PSA. Clin. Radiol..

[13] Futterer J.J., Briganti A., De Visschere P., Emberton M., Giannarini G., Kirkham A., Taneja S.S., Thoeny H., Villeirs G., Villers A. (2015). Can Clinically Significant Prostate Cancer Be Detected with Multiparametric Magnetic Resonance Imaging? A Systematic Review of the Literature. Eur. Urol..

[14] Barentsz J.O., Richenberg J., Clements R., Choyke P., Verma S., Villeirs G., Rouviere O., Logager V., Futterer J.J. (2012). ESUR prostate MR guidelines 2012. Eur. Radiol..

[15] Weinreb J.C., Barentsz J.O., Choyke P.L., Cornud F., Haider M.A., Macura K.J., Margolis D., Schnall M.D., Shtern F., Tempany C.M. (2016). PI-RADS Prostate Imaging-Reporting and Data System: 2015, Version 2. Eur. Urol..

[16] Barentsz J.O., Weinreb J.C., Verma S., Thoeny H.C., Tempany C.M., Shtern F., Padhani A.R., Margolis D., Macura K.J., Haider M.A. (2016). Synopsis of the PI-RADS v2 Guidelines for Multiparametric Prostate Magnetic Resonance Imaging and Recommendations for Use. Eur. Urol..

[17] Richards C.B., Corfield A.B., Cleaveland P., Tang V.C., Sinclair A.N., Dyer J.E. (2023). Evaluation of Patients Referred for Abnormal Digital Rectal Examination With Normal Prostate-Specific Antigen on Best Timed Pathway for Prostate Cancer. Cureus.

[18] Al-Monajjed R., Schimmoller L., Radtke J.P., Lakes J., Krilaviciute A., Schlemmer H.P., Herkommer K., Seibold P., Becker N., Kaaks R. (2025). Characterisation of Multiparametric Magnetic Resonance Imaging of the Prostate in Younger Men with Normal Prostate-specific Antigen Within the PROBASE Study. Eur. Urol. Open Sci..

[19] De Visschere P., Oosterlinck W., De Meerleer G., Villeirs G. (2010). Clinical and imaging tools in the early diagnosis of prostate cancer, a review. J. Belg. Soc. Radiol..

[20] Schroder F.H., van der Maas P., Beemsterboer P., Kruger A.B., Hoedemaeker R., Rietbergen J., Kranse R. (1998). Evaluation of the digital rectal examination as a screening test for prostate cancer. Rotterdam section of the European Randomized Study of Screening for Prostate Cancer. J. Natl. Cancer Inst..

[21] Krilaviciute A., Becker N., Lakes J., Radtke J.P., Kuczyk M., Peters I., Harke N.N., Debus J., Koerber S.A., Herkommer K. (2023). Digital Rectal Examination Is Not a Useful Screening Test for Prostate Cancer. Eur. Urol. Oncol..

[22] Matsukawa A., Yanagisawa T., Bekku K., Kardoust Parizi M., Laukhtina E., Klemm J., Chiujdea S., Mori K., Kimura S., Fazekas T. (2024). Comparing the Performance of Digital Rectal Examination and Prostate-specific Antigen as a Screening Test for Prostate Cancer: A Systematic Review and Meta-analysis. Eur. Urol. Oncol..

[23] Sajjad W., Thankappannair V., Shah S., Ahmed A., Saeb-Parsy K., Kastner C., Lamb B., Gnanapragasam V.J. (2024). Diagnostic value of the abnormal digital rectal examination in the modern MRI-based prostate cancer diagnostic pathway. J. Clin. Urol..

[24] Han E., Ho J., Choi W., Yoo I., Chung S. (2010). Significance of incidental focal uptake in prostate on 18-fluoro-2-deoxyglucose positron emission tomography CT images. Br. J. Radiol..

[25] Marsden T., Ahmed H.U., Emberton M., Re I.S.G. (2021). An update from the ReIMAGINE Prostate Cancer Risk Study (NCT04060589): A prospective cohort study in men with a suspicion of prostate cancer who are referred onto a magnetic resonance imaging-based diagnostic pathway with donation of tissue, blood, and urine for biomarker analyses. Eur. Urol..

[26] Messina E., La Torre G., Pecoraro M., Pisciotti M.L., Sciarra A., Poscia R., Catalano C., Panebianco V. (2024). Design of a magnetic resonance imaging-based screening program for early diagnosis of prostate cancer: Preliminary results of a randomized controlled trial-Prostate Cancer Secondary Screening in Sapienza (PROSA). Eur. Radiol..

[27] Nam R.K., Wallis C.J., Stojcic-Bendavid J., Milot L., Sherman C., Sugar L., Haider M.A. (2016). A Pilot Study to Evaluate the Role of Magnetic Resonance Imaging for Prostate Cancer Screening in the General Population. J. Urol..

[28] Kohestani K., Mansson M., Arnsrud Godtman R., Stranne J., Wallstrom J., Carlsson S., Hellstrom M., Hugosson J. (2021). The GOTEBORG prostate cancer screening 2 trial: A prospective, randomised, population-based prostate cancer screening trial with prostate-specific antigen testing followed by magnetic resonance imaging of the prostate. Scand. J. Urol..

[29] Schouten M., van der Leest M., Pokorny M., Hoogenboom M., Barentsz J., Thompson L., Fütterer J. (2017). Why and where do we miss significant prostate cancer with multi-parametric magnetic resonance imaging followed by magnetic resonance-guided and transrectal ultrasound-guided biopsy in biopsy-naïve men?. Eur. Urol..

[30] Chatterjee A., Gallan A., Fan X., Medved M., Akurati P., Bourne R., Antic T., Karczmar G., Oto A. (2023). Prostate Cancers Invisible on Multiparametric MRI: Pathologic Features in Correlation with Whole-Mount Prostatectomy. Cancers.

